# Bilateral acute multifocal retinitis and retinal vasculitis secondary to Rickettsia typhi infection

**DOI:** 10.1186/s12348-025-00496-4

**Published:** 2025-04-21

**Authors:** Weilin Song, Adrian Au, David Sarraf, Pradeep Prasad, Edmund Tsui

**Affiliations:** 1 Stein Eye Institute, Department of Ophthalmology, University of California Los Angeles, 100 Stein Plaza, Los Angeles, CA 90095 USA; 2 Greater Los Angeles VA Healthcare Center, Los Angeles, CA USA

**Keywords:** Acute multifocal retinitis, Rickettsia, Typhus

## Abstract

**Purpose:**

To describe a case of acute multifocal retinitis (AMR) and retinal vasculitis associated with *Rickettsia typhi*.

**Methods:**

Case report.

**Results:**

A 37-year-old previously healthy female presented with acute bilateral panuveitis that was preceded by a febrile illness with pneumonitis and transaminitis. On exam she had bilateral multifocal small white retinal lesions, vascular sheathing, and hemorrhages. The retinal lesions, which appeared consistent with infiltrates and/or ischemia, were confined within the inner or middle retinal layers on optical coherence tomography (OCT) and corresponded to late leakage on fluorescein angiography (FA). There was no evidence of choroidal involvement on indocyanine green angiography (ICGA). Based on the imaging findings and history, the diagnosis of AMR with associated small vessel retinal vasculitis was made and the patient was started empirically on doxycycline. Workup was positive for *R. typhi*. At follow-up, there was resolution of visual symptoms and nearly all retinal lesions.

**Conclusions:**

Rickettsial disease should be highly suspected in a patient with AMR and occlusive small vessel vasculitis. Retinal lesions may be either infiltrative or ischemic in nature. Diagnosis, which can be aided by multimodal retinal imaging, is essential for prompt initiation of appropriate antibiotic therapy.

## Introduction

Acute multifocal retinitis (AMR) is a rare condition characterized by multiple small or mid-sized superficial lesions localized to the inner retina. AMR affects otherwise young healthy adults and is preceded by a flu-like viral prodrome that resolves over weeks to months, with visual recovery. While many cases are idiopathic, AMR can also be associated with rickettsia, cat-scratch disease, syphilis, and Q fever [1–4]. 

*Rickettsia typhi* belongs to the typhus group of the *Rickettsia* genus and is characterized by gram negative, obligate intracellular, rod-shaped bacteria. Infections manifest clinically in the initial stages as an acute, nonspecific, febrile illness, and transmission occurs by multiple vectors including ticks, fleas, lice, and mites [5]. While typhus can occur worldwide, *R. typhi* thrives best in coastal areas. Within the United States, cases of typhus are becoming increasingly more prevalent in endemic regions including California, Texas, and Hawaii [6]. However, it is estimated that fewer than a third of flea-borne typhus cases are ever diagnosed, in part due to nonspecific systemic symptoms and the lack of an accurate, rapid diagnostic test [7]. Rickettsial infection is also underestimated as a cause of infectious uveitis in the literature [8], perhaps due to its self-limiting course, but may be a common cause of AMR [2]. 

While retinal lesions in AMR are thought to be solely infiltrative in nature, we report a case of R. typhi associated AMR and small vessel retinal vasculitis with both infiltrative and ischemic lesions within the inner and middle retina on multimodal imaging.

## Case

A 37-year-old healthy white female presented with acute onset bilateral floaters, photopsias and blurred vision for one day. Two weeks prior, she developed respiratory symptoms, fevers and diarrhea. Workup was notable for ground-glass opacities and multifocal pneumonia on chest x-ray for which she was treated with azithromycin, and elevated liver enzymes consistent with transaminitis. Social history was notable for recent exposure to kittens but no recent travel or known tick bites. Past medical and ocular histories were unremarkable.

**Fig. 1:**
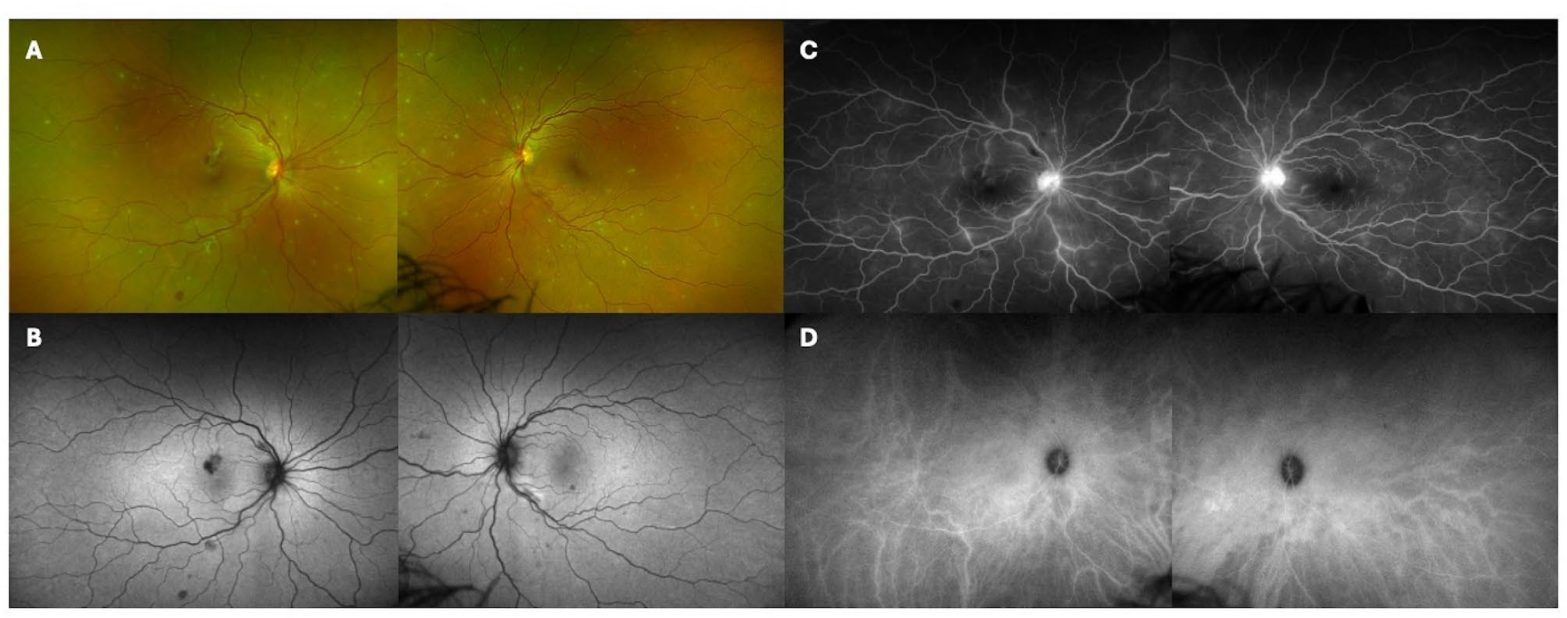
Ultra-widefield color photos at presentation of the right and left eyes show multifocal retinal infiltrates with scattered areas of intraretinal hemorrhage (**A**). Fundus autofluorescence photos of the right and left eyes show hypoautofluorescence corresponding to blockage from retinal hemorrhage (**B**). Fluorescein angiography of the right and left eyes shows optic disc leakage and late vascular leakage (**C**). Indocyanine green angiography of the right and left eyes at 10 minutes do not demonstrate any choroidal lesions (**D**)

On exam, her visual acuity was 20/30 in the right eye and 20/25 in the left eye. Anterior segment exam was remarkable for 2 + anterior chamber cell and 2 + vitreous cell in both eyes. Fundus exam was notable for multifocal small yellow retinal lesions in the posterior pole with extension to the nasal periphery in each eye (Fig. 1A). Retinal vessel tortuosity with subtle sheathing was noted in each eye and bilateral scattered intraretinal hemorrhages were more prominent in the right eye (Fig. 1A). Fundus autofluorescence imaging demonstrated hypoautofluorescence corresponding to the hemorrhages and variable hypoautofluorescence corresponding to the retinal lesions (Fig. 1B). On fluorescein angiography (FA), there was bilateral optic disc leakage and late retinal vascular leakage corresponding to retinal lesions but noareas of non-perfusion (Fig. 1C). Indocyanine green angiography (ICGA) excluded choroidal involvement (Fig. 1D).

**Fig. 2:**
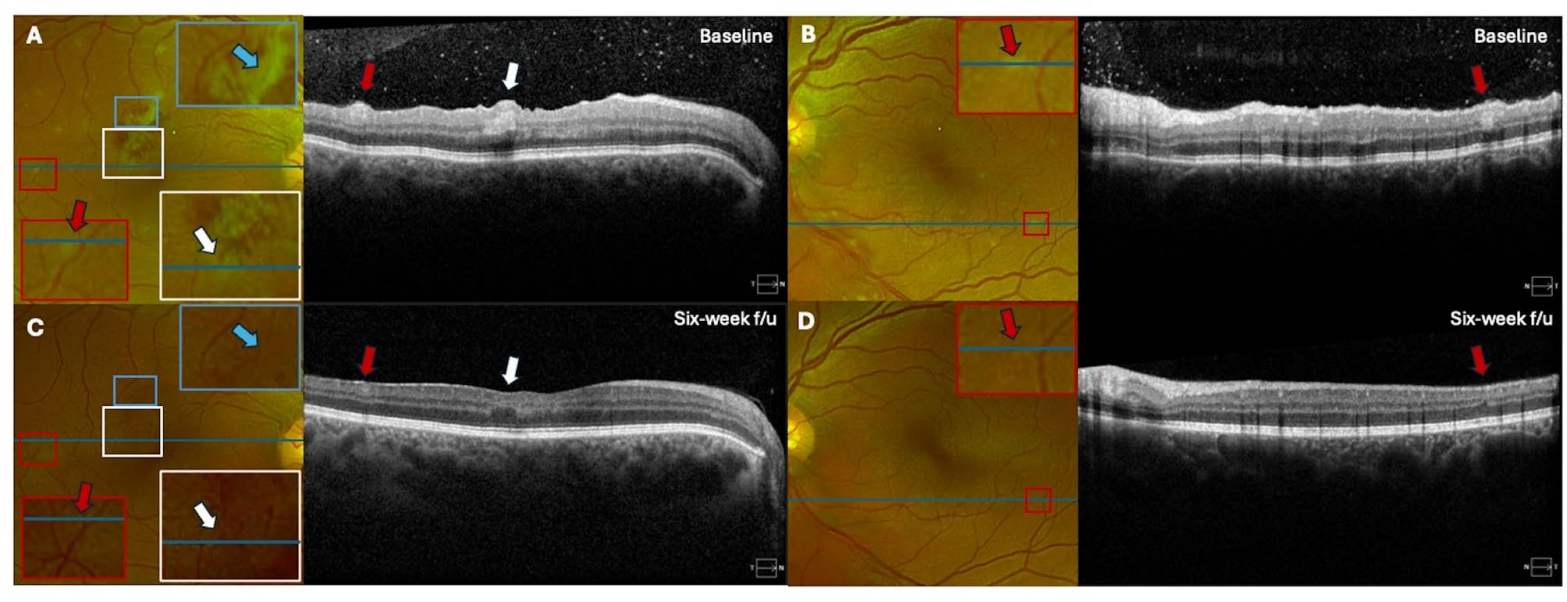
Baseline optical coherence tomography (OCT) B scans of the right eye show a focal area of arteritis, middle retinal hyperreflectivity, and ischemia consistent with paracentral acute middle maculopathy (PAMM) (**A**, white arrows) with associated vascular sheathing on color fundus imaging (**A**, blue arrows). There is also a focal area of inner retinal thickening (**A**, red arrows). OCT B scans of the left eye show focal area of middle retinal thickening (**B**, red arrows). (**B**) At follow up six weeks later, OCT B scans through the macula and through retinal lesions show nearly complete resolution of the retinal lesions (**C**, **D**; red arrows), with residual inner and middle retinal thinning and infarction consistent with a retinal ischemic perivascular lesion (RIPL) in the right eye (**C**; white arrow)

On optical coherence tomography (OCT) imaging in the right eye, there was a focal region of arteritis with middle retinal ischemia consistent with paracentral acute middle maculopathy (PAMM) (Fig. 2A, white arrows) that was associated with vascular sheathing on fundus imaging (Fig. 2A, blue arrows). OCT imaging through a peripheral retinal lesion in the right eye also demonstrated focal inner retinal thickening that appeared consistent with an infiltrative process (Fig. 2A, red arrow). In the left eye, OCT imaging through a peripheral retinal lesion demonstrated focal middle retinal thickening that may have reflected either an infiltrative or ischemic process (Fig. 2B, red arrows).

Based on the clinical presentation and multimodal imaging, a diagnosis of AMR was suspected, and the patient was treated empirically with oral doxycycline 200 mg daily for 14 days along with prednisolone acetate 1% eye drops four times a day for one week then tapered by one drop every two weeks. Infectious and inflammatory workup, including serum rapid plasma regain (RPR) and Treponema pallidum particle agglutination (TP-PA), toxoplasmosis IgG/IgM, Bartonella IgG/IgM, Rickettsia IgG/IgM/Titer, West Nile IgG/IgM, QuantiFERON-Gold, ANCA, anti-nuclear antibody (ANA), angiotensin converting enzyme (ACE), lysozyme, chest x-ray and blood bacterial and fungal cultures, were negative except for rickettsial testing with positive IgM and IgG titers for *R. typhi*.

**Fig. 3:**
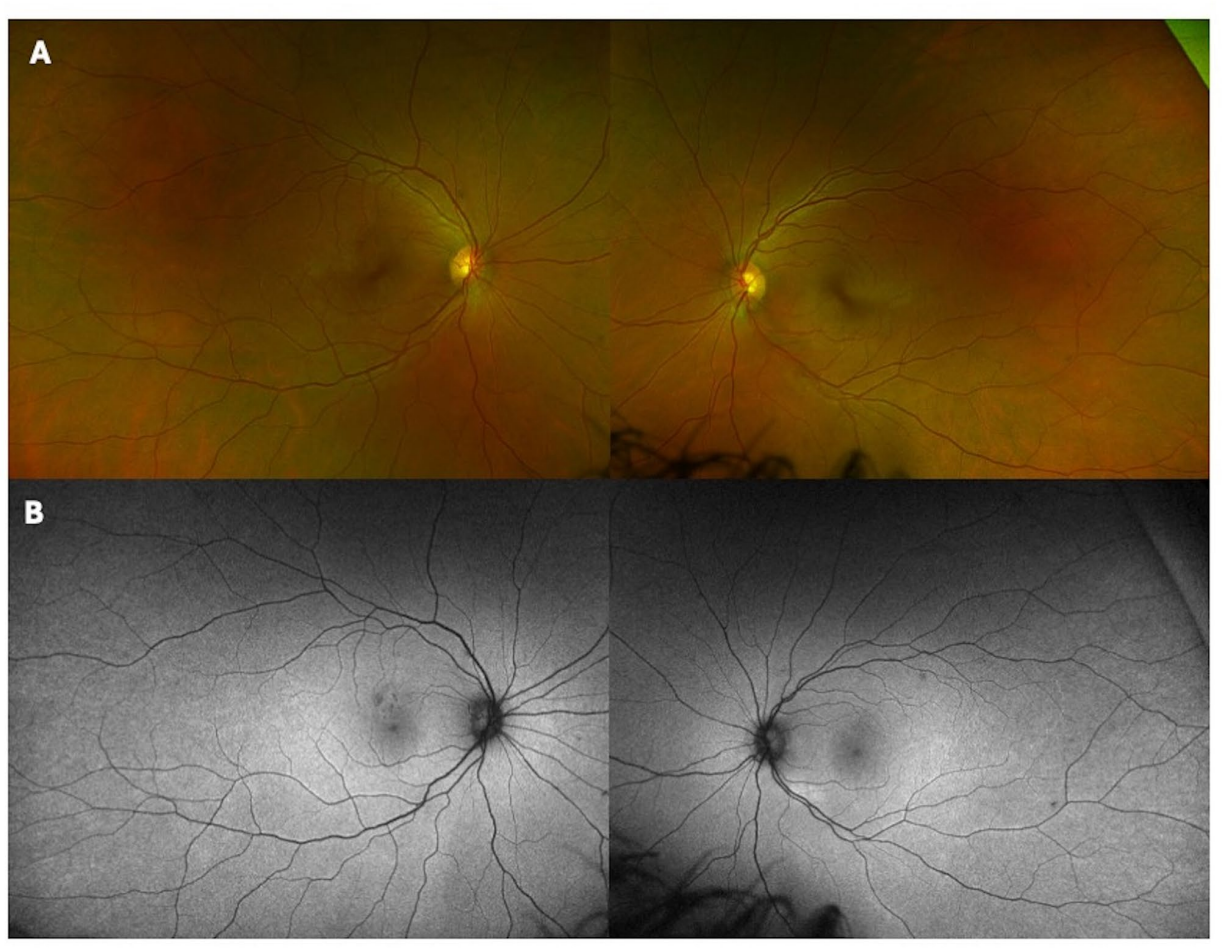
Ultra-widefield color fundus (**A**) and autofluorescence photos (**B**) of the right and left eye show nearly complete resolution of multifocal retinal infiltrates and hemorrhages at follow up six weeks later

One month after completing doxycycline, the patient’s visual acuity improved to 20/20 bilaterally with symptom resolution. On exam, the anterior and posterior inflammation, yellow inner and middle retinal lesions (red arrows) and intraretinal hemorrhages were resolved (Figs. 2C-D and 3A-B). The PAMM lesion in the right eye evolved to a small area of middle retinal thinning consistent with retinal ischemic perivascular lesion (RIPL) (Fig. 2C; white arrow).

## Discussion

In summary, this report describes an otherwise healthy female patient who developed acute vision loss after developing a febrile illness with pneumonitis and transaminitis. Examination was notable for anterior chamber inflammation, vitritis, bilateral multifocal retinal lesions, and vascular sheathing consistent with AMR and associated small vessel retinal vasculitis. Comprehensive infectious and inflammatory workup was completed and was positive for *R. typhi*.

The differential diagnosis of inflammatory multifocal inner retinal lesions includes infectious etiologies such as rickettsia, syphilis, Bartonella, Lyme disease, and some mosquito-borne viral diseases [1, 9–11]. While the majority of published AMR cases are classically thought to be idiopathic, many of the prior reported cases did not include rickettsial serology and the appropriate diagnosis may have been missed [1, 3, 4, 9, 12, 13]. In one case series of 35 patients (60 eyes) with AMR, Mediterranean spotted fever (*R. conorii*) was positive in 20 patients (57%) and murine typhus (*R. typhi*) in 4 patients (11%).^2^ More recently, a case of bilateral retinochoroiditis and vitritis secondary to *R. typhi* infection was reported, unlike our case without choroidal involvement [14]. 

The diagnosis of AMR was made in this case due to localization of retinal infiltrates to the inner retina on OCT with no outer retinal layer involvement [3]. While there was no obvious evidence of choroidal involvement with ICGA in this patient, subclinical hypofluorescent lesions visible only on ICGA have been previously described in a subset of patients with serology-proven murine typhus and AMR [15]. There was no optic disc swelling or exudative retinal detachment, which have been previously reported to complicate AMR [1, 3, 4, 9, 12, 13]. In addition to infiltrates, the retinal lesions may also reflect middle retinal ischemia secondary to concurrent occlusive small vessel retinal vasculitis. Rickettsial infection has been reported to be a leading cause of inflammatory branch retinal artery occlusion, and incidences of central retinal artery occlusion and retinal vein occlusions have also been reported [8, 16, 17]. While the majority of retinal infiltrates of AMR completely resolve spontaneously or with antibiotic treatment; [1, 2, 9, 13] a subset of eyes develop residual localized inner retinal layer defects that may be a sequalae of ischemia [2]. Our patient was noted to have a focal region of inner and middle retinal thinning and infarction (as a legacy of PAMM) consistent with a retinal ischemic perivascular lesion (RIPL) in the right eye on follow-up [18]. Localized retinal nerve fiber layer (RNFL) defects with corresponding visual field defects have also been reported as a sequelae of superficial retinal infiltrates in inflammatory and infectious conditions such as Behcet uveitis, ocular toxoplasmosis, and chikungunya virus infections [19]. While there does not appear to be a localized RNFL defect in our patient on follow-up, RNFL thickness analysis and visual field testing can be considered to rule out chronic sequalae with visual consequences.

The exact pathophysiology of AMR remains to be elucidated. Focal retinal infiltrates associated with rickettsial infection, cat-scratch disease, or syphilis may result directly from intraretinal bacterial multiplication, or from immune-mediated response to bacterial antigens caused by the deposition of immune complexes, inflammatory cells, or antibodies [1, 20]. Rickettsia preferentially infects the vascular endothelial cells lining the small and medium vessels throughout the body, which may explain the associated small vessel retinal vasculitis and ischemic lesions observed in this case [21]. Whether the retinal lesions are predominantly ischemic or infiltrative requires further investigation.

Like other rickettsial illnesses, *R. typhi* infection is associated with a diverse range of nonspecific presentations from mild fever and rash to severe sepsis with multiorgan involvement [22]. Serology to detect IgG and IgM antibodies is the mainstay for laboratory diagnosis, although there are significant limitations. Serological evidence of rickettsial infection does not become apparent until the second week of disease, therefore, a nonreactive or low-titer result does not exclude the diagnosis if the testing was done within the first seven days of illness [23, 24]. Given the significant association with rickettsial infection, patients presenting with AMR and nonspecific systemic symptoms should be started on empiric antibiotic therapy before serologic confirmation. While the prognosis of rickettsial infection is good in most cases, severe and life-threatening complications including severe interstitial pneumonitis, meningoencephalitic syndrome, acute renal failure, myocarditis, and disseminated intravascular coagulation may occur and can be prevented by early antibiotic therapy [8, 23, 25]. 

Our patient developed pneumonitis and transaminitis prior to her ocular symptoms, which are associated systemic features of *R. typhi* infection [26, 27]. Situ et al. similarly reported two cases of uveitis, characterized by multifocal white spots within the retina, with severe transaminitis associated with *R. typhi* infection [28]. However, OCT imaging was not provided to demonstrate that the lesions were inner retinal. Both patients were initially treated with oral prednisone and did not receive doxycycline until serology was positive for *R. typhi*.

In conclusion, given its association with both presentations, rickettsial illness should be highly suspected in patients with AMR and associated occlusive small vessel retinal vasculitis– appropriate serology should be ordered and empiric antibiotic therapy should be started. Multimodal imaging is a helpful diagnostic aid for AMR, a rare presentation of uveitis that classically was considered a diagnosis of exclusion. Early clinical diagnosis of rickettsial illness is of utmost importance for prompt initiation of appropriate antibiotic therapy to facilitate retinal and visual recovery and to decrease systemic illness severity and duration.

## Data Availability

No datasets were generated or analysed during the current study.
